# High-Throughput Screening of Lipidomic Adaptations in Cultured Cells

**DOI:** 10.3390/biom9020042

**Published:** 2019-01-24

**Authors:** Aike Jeucken, Jos F. Brouwers

**Affiliations:** 1 Membrane Enzymology, Groningen Biomolecular and Biotechnology Institute (GBB), University of Groningen, 9747AG Groningen, The Netherlands; a.jeucken@rug.nl; 2 Department of Biochemistry and Cell Biology, Faculty of Veterinary Medicine, Utrecht University, 3584CM Utrecht, The Netherlands

**Keywords:** lipidomics, autophagy, fatty acid synthase, high-throughput, phospholipids, lipid metabolism

## Abstract

High-throughput screening of biologically active substances in cell cultures remains challenging despite great progress in contemporary lipidomic techniques. These experiments generate large amounts of data that are translated into lipid fingerprints. The subsequent visualization of lipidomic changes is key to meaningful interpretation of experimental results. As a demonstration of a rapid and versatile pipeline for lipidomic analysis, we cultured HeLa cells in 96-well format for four days in the presence or absence of various inhibitors of lipid metabolic pathways. Visualization of the data by principle component analysis revealed a high reproducibility of the method, as well as drug specific changes to the lipidome. Construction of heatmaps and networks revealed the similarities and differences between the effects of different drugs at the lipid species level. Clusters of related lipid species that might represent distinct membrane domains emerged after correlation analysis of the complete dataset. Taken together, we present a lipidomic platform for high-throughput lipidomic analysis of cultured cell lines.

## 1. Introduction

High-throughput lipidomics has great potential when investigating the role of lipids in cellular metabolism. A large number of drugs is available to interfere with normal lipid metabolic pathways and contemporary lipidomic techniques can monitor the levels of hundreds of lipids simultaneously. However, true high-throughput lipidomics still involves the subsequent overcoming of several challenges. First, the clean-up of samples so their lipid composition may be measured. Established protocols for extraction of lipids involve liquid-liquid extractions after an initial one-phase system [1,2,3]. In this one phase system, lipids and hydrophilic metabolites remain dissolved, whereas proteins precipitate and are removed by centrifugation. The subsequent induction of a two-phase system by the addition of water and/or organic solvent, separates hydrophilic- and hydrophobic metabolites. Together with additional washing steps to increase lipid recovery, the two-phase extraction process is laborious, time-consuming, and cumbersome to automate.

Next, a choice has to be made between shotgun lipidomics or a liquid chromatography-mass spectrometry (LC-MS^n^) approach. Shotgun lipidomics, i.e., the direct infusion of the lipid extract, relies solely on mass spectrometry techniques for lipid fingerprinting [4,5,6]. The LC-MS^n^ approaches based on either reversed phase- (RP), normal phase- (NP), or hydrophilic interaction liquid chromatography (HILIC) have all proven their value as they add retention time as an additional feature that can aid in the identification of lipid species (reviewed in [7,8,9]). In particular HILIC based separation of lipid classes allows for LCMS based lipidomic analysis in time-spans normally only achievable by shotgun lipidomics [10,11]. Here, we used such a rapid HILIC-LCMS technique to obtain maximum sensitivity and specificity, while avoiding the ion suppression commonly associated with shotgun lipidomics.

For the translation of LC-MS data to a (semi-)quantitative and annotated peak list, several strategies have been successfully demonstrated for high- as well as low resolution instruments [12,13,14,15,16]. Typically, a lipidomics experiment results in the identification of several hundreds of lipid species. The interpretation of the obtained results is more challenging than in other -omics fields. For instance, in proteomics and genomics experiments, the changing proteins may be directly linked to enzymatic or signaling pathways, thus giving clear clues to altered cellular functions. Also in (hydrophilic) metabolomics, most metabolites are the product of one or very few enzymes, and are a substrate for very few other enzymes. Therefore, also in metabolomics, altered levels of metabolites can be directly mapped to changes in metabolic pathways. In lipidomics, this mapping is more challenging, but bioinformatics have become an integral part of the lipidomic pipeline [17,18].

Lipidomic data contain an additional layer of detail. Whereas the two-dimensional (2D) metabolic maps typically present a lipid class such as phosphatidylcholine (PC) or phosphatidylethanolamine (PE) as a single metabolite, the lipid subclass and acyl composition add a third dimension to the map. Lipid functions can be dependent on this additional layer, for instance phospholipases may act only on a subset of lipid species within a lipid class and the occurrence of lipid microdomains is also regulated at the lipid species level [19,20,21]. Interpretation of lipidomic data, therefore, is particularly challenging.

Likewise, interference with lipid metabolism, be it drug induced or resulting from pathology, is likely to affect many lipid species. Discriminating between key primary effects and less relevant side-effects is impossible without considering the entire (phospho-)lipidome and understanding existing interactions between lipid species. Therefore, information-rich visualizations of the changes in the complex lipidome are important. Once this is achieved, multiple drugs or cellular conditions may be compared (see [22] for examples of such high-content visualizations).

Here, we compare the effects of nine different drugs on the cellular lipidome of the HeLa cell line. We chose drugs that are widely used in literature, are readily available and target a variety of cellular processes. These experiments were performed using a high-throughput approach, which enables a scientist to complete the entire lipidomic analysis from lipid extraction and LC-MS to visualizations within a single day. In this way, we could demonstrate a high reproducibility of the lipidomic changes in our experiments. Furthermore, we visualize similarities and differences between drugs and their effects on lipid species. Our use of inhibitors that interfere at different points in lipid metabolism, enabled us to investigate which lipid species strongly correlate to each other, either positively or negatively. Taken together, we demonstrate how lipidomics can be implemented in high content-omics screens.

## 2. Materials and Methods

### 2.1. Chemicals

Dulbecco’s modified Eagle’s medium (DMEM) and fetal bovine serum (FBS) were obtained from Gibco (Paisly, United Kingdom). Inhibitors and dimethyl sulfoxide (DMSO) were obtained from Sigma (city, state if USA, country). Methanol, acetonitrile, acetone, and ammonium formate were purchased from BioSolve (Valkenswaard, The Netherlands), chloroform was obtained from Roth (Karlsruhe, Germany) and were all HPLC/MS grade.

### 2.2. Cell Culture

HeLa cells were plated at a density of 2 × 10^3^ cells/well in glass coated 96-wells plates (Thermofisher Scientific, Waltham, MA, catalogue number 60180-P304). Inhibitors, dissolved in DMSO, were added to obtain the following final concentrations; 1.25 μM myriocin, 100 μM rosiglitazone, 25 μM bafilomycin, 40 μM orlistat, 10 μM spautin, 50 μM etomoxir, 20 μM C75, 10 μM celecoxib, 20 μM fumonisinB1, for the control only the same amount of DMSO was added (0.1%, *v/v*). Cells were cultured in DMEM supplemented at the start with 10% FBS in a humidified 5% CO_2_ incubator at 37 °C for four days. Cell growth and survival were verified by assessment of phospholipid content of wells after washing (see below).

### 2.3. Lipid Extractions

Cells were washed once with cold phosphate-buffered saline solution (PBS) to remove medium and dead cells, followed by resuspending in 150 µL chloroform/methanol (1:1 *v/v*) and lipids were extracted for 1 h at 4 °C, followed by centrifugation (1800 g, 20 min, 4 °C) to remove proteins and other macromolecules. The supernatant was transferred to a new plate that was then covered by aluminum foil to prevent evaporation of organic solvents. From this plate, 10 µL aliquots were taken by an autosampler for LC-MS.

### 2.4. Liquid Chromatography-Mass Spectrometry 

The phospholipid species composition was determined by liquid chromatography coupled to mass spectrometry (LC-MS). Instrumentation used consisted either of an Infinity II 1290 UPLC (Agilent, Santa Clara, CA, USA) coupled to an Orbitrap Fusion (see below), or consisted of a Dionex HPG-3200RS UPLC (ThermoFisher Scientific, Waltham, MA, USA) coupled to a LTQ-XL (see below). The extracted lipids were loaded on a HILIC column (2.6 µm HILIC 100 Å, 50 × 4.6 mm, Phenomenex, Torrance, CA, USA) maintained at 25 °C and eluted at a flow rate of 1 mL/min with a gradient from acetonitrile/acetone (9:1, *v/v*) to acetonitrile/H_2_O (7:3, v/v) with 10 mM ammonium formate. Both elution solutions also comprised 0.1% (*v/v*) formic acid. The column outlet of the LC was connected either to a heated electrospray ionization (HESI) source of an Orbitrap Fusion mass spectrometer or the atmospheric pressure chemical ionization source of an LTQ-XL mass spectrometer (ThermoFisher Scientific). Full scan spectra were collected either in negative ionization mode (Fusion) or in positive ionization mode (LTQ-XL). Mass spectrometer parameter settings are listed in the supplementary methods file. Data dependent MS2 was performed in parallel with Orbitrap MS1 scanning on the Fusion mass spectrometer and was used for lipid class confirmation only.

### 2.5. Software and Bioinformatics

The LC-MS data were converted to mz(X)ML format by msconvert from the ProteoWizard toolbox, using vendor peak picking. Data was analyzed using XCMS version 1.52.0 running under R version 3.4.3 and included peak grouping, peak alignment, and the forced integration of missed peaks [23,24]. Peak integration and alignment parameters are given in the supplementary methods file. Prior to statistical analysis, annotated peak lists were normalized to 100% total peak area. Principle component analysis provided by the R package pcaMethods was used to visualize the multidimensional LC-MS data [25].

## 3. Results

### 3.1. Profiling of the HeLa Cell Lipidome

**Figure 1:**
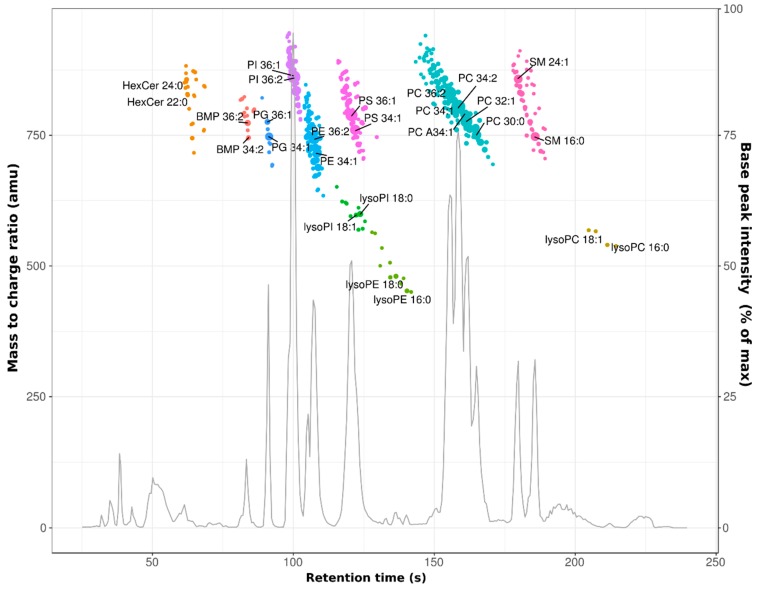
Combined base peak chromatogram and contour plot of HeLa lipids during exponential cell growth. Cells were lysed in chloroform / methanol and the resulting extract was injected directly on the high-performance liquid chromatography (HPLC) column after removal of precipitated proteins and macro-molecules by centrifugation

A total of 249 lipids were identified with atmospheric pressure chemical ionization (APCI) LC-MS by linear ion trap MS (Table S1). Compared to the more commonly used electrospray ionization, APCI has the advantage that it has more similar response factors for the various lipid classes in the positive ionization mode [7]. On the downside, APCI is less sensitive than electrospray and lipid headgroups are lost due the harsher ionization conditions. Furthermore, low resolution and low mass-accuracy instruments such as linear ion traps, cannot discriminate between nominally isobaric diacyl- and ether lipid species by MS1 alone. To validate the ion trap data and discriminate between isobaric species where possible, we also analyzed the lipidome of HeLa cells grown in the absence of any inhibitor by ultrahigh resolution mass spectrometry. This resulted in the identification of 451 phospholipid species in negative ionization mode, using formate adducts for sphingomyelin and (lyso-) phosphatidylcholine (Figure 1 and Table S2). The retention time difference of ~1 s between nominally isobaric species (see retention times in Table S2), were often too small for unequivocal assignment of low-resolution MS data. In these cases, the total lipid signal was annotated with the name of the most abundant species in the ultrahigh resolution analysis. Neutral lipids such as triacylglycerols, cholesterol and cholesterol esters, eluted within the first 30 s of the run. However, due to ion suppression by background ions, poor ionization efficiency of neutral lipids in (negative mode) HESI and their very narrow elution windows, they could not be reliably quantified.

### 3.2. Inhibitors Induce Specific Alterations of HeLa Lipidomes

**Figure 2:**
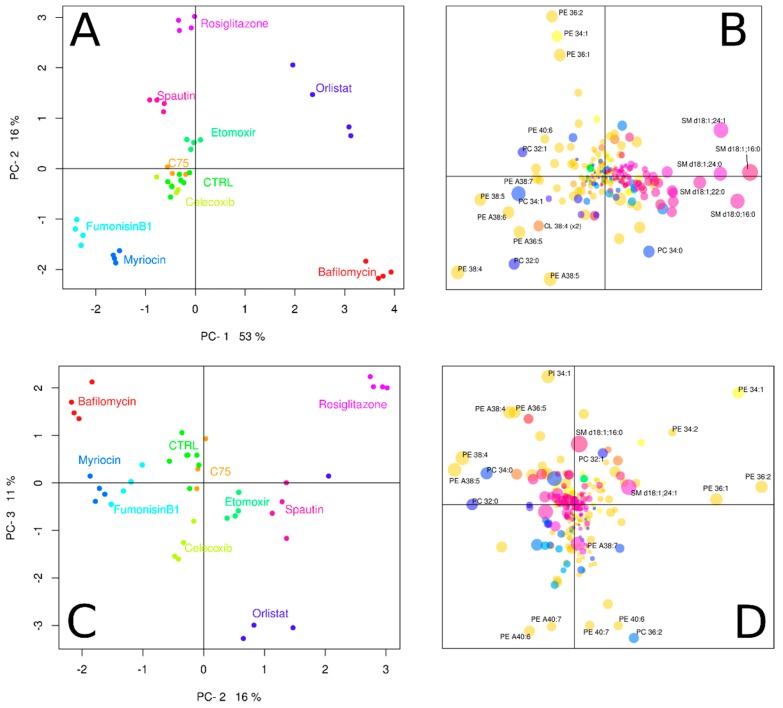
Distinct lipidomic changes are induced by the presence of inhibitors. (**A**) Principal component analysis (PCA) score plot for the different lipidomes (PC-1 and PC-2). The CTRL indicates that only DMSO was added to the growth medium (green), bafilomycin (red), C75 (orange), celecoxib (spring green), etomoxir (light green), fumonisinB1 (cyan), myriocin (light blue), orlistat (dark blue), rosiglitazone (purple), spautin (pink). (**B**) PCA loading plot (PC-1 and PC-2), lipid species that contributed the most to the variance are labeled, the size of the dots indicates relative abundance. (**C**) Principal component analysis score plot for PC-2 and PC-3. (**D**) PCA loading plot (PC-2 and PC-3). Sphingosine backbones were assumed for sphingomyelin (SM) species. An ‘A’ in the lipid name indicates the presences of an ether linkage.

Subsequently, the obtained lipidomic data from HeLa cells grown in the presence absence of the various drugs, were visualized with principal component analysis (PCA). In the PCA score plot (Figure 2A), similar lipidomes map closely together. Nearly all HeLa cells grown in the presence of an inhibitor, resulted in lipidomes that were distinct from those grown under control conditions. The four parallel cultures that were grown under identical conditions had very reproducible lipid compositions, as can be concluded from the excellent clustering of these samples in Figure 2A. Addition of celicoxib or C75 did not seem to have an effect on the cell line’s lipidome, as these incubations overlap with the control (CTRL). It should be noted that Figure 2A only represents 69% of the total variance in the lipidomic data set; 53% in principal component 1 and 16% in principal component 2. Inclusion of principal component 3, reflecting an additional 11% of the dataset variance, then revealed that celicoxib actually had a non-overlapping lipidome with the control (Figure 2C). Additional principal components beyond PC-3 did not reveal further drug-induced lipidomic changes (data not shown). Therefore, it should be concluded that only C75 did not induce lipidomic changes under our experimental conditions.

**Figure 3:**
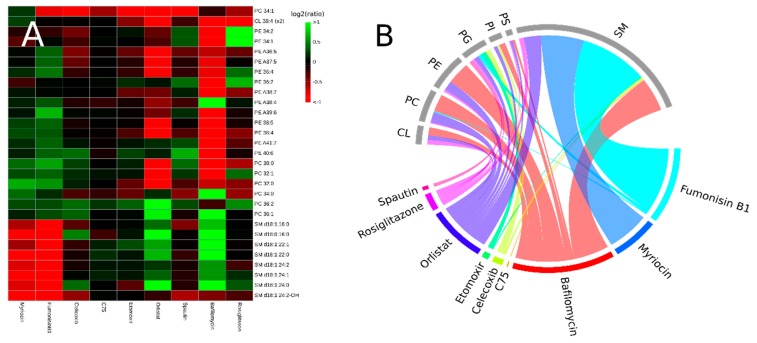
Correlations between inhibitors and lipid species, and connections between inhibitors and lipid classes. (**A**) A heatmap of lipids species that at least contribute to 0.5% of the lipidome of the control samples, and that are changed at least by a factor 2 (up or down) with a *p* < 0.001. (**B**) A chord diagram showing connections between inhibitors and lipid classes, the width of the chords is determined the number of lipid species that significantly (*p* < 0.001) changed upon addition of the inhibitor.

To identify lipid species of special interest that changed during any of the experimental conditions, we constructed a heatmap from lipids and inhibitors (Figure 3A). In this heatmap, we only included species that changed at least by a factor two (up or down) compared to the control in at least one of the experimental conditions. We then considered species that did not contribute at least 0.5% to the total area of the lipid signals in the LC-MS data of the control samples, to be of minor biological importance in these experiments. Finally, species that met these two criteria, were only included if *p* < 0.001 compared to control, to correct for false positives due to multiple testing. Details of this heatmap are discussed below, together with the corresponding inhibitor. A visualization of all interactions with *p* < 0.001 between inhibitors and phospholipid classes is depicted in Figure 3B.

#### 3.2.1. Interference with Sphingolipid Biosynthesis by Myriocin and Fumonisinb1

Fumonisin B1 and myriocin are both natural products that are synthesized by fungi. Due to their structural analogy to sphinganine/sphingosine, they act as competitive inhibitors of the sphingolipid biosynthetic pathway [26,27]. In our experiments, myriocin and fumonisinB1 reduced the levels of sphingomyelin (SM) by 41% (*p* < 10^-7^) and 60% (*p* < 10^-9^), respectively. The PCA loading plot (Figure 2B) indicates that these two inhibitors affect all SM species. Sphingomyelin species have a positive loading on PC-1 (they are at the right half of the loading plot), but the two inhibitors have negative PC-1 scores. The (mechanistic) similarity of the two sphingolipid inhibitors is further illustrated by their relative proximity in the lipidomics landscape as outlined in the PCA score plot (Figure 2A). From the heatmap in Figure 3A, it can be concluded that the reduction in SM content was compensated by a variety of species from other lipid classes (green squares). Notably, there was a clear difference between the two inhibitors in which lipid species contributed most to this compensation, demonstrating that these inhibitors are not interchangeable in lipidomic experiments. Nevertheless, both inhibitors specifically target SM species (Figure 3B).

#### 3.2.2. Celicoxib, a cyclooxigenase-2 Inhibitor

Celicoxib is a non-steroidal anti-inflammatory drug that acts specifically on the cyclooxygenase-2 (COX-2). COX-2 is best known as an inducible protein, expressed at sites of inflammation, infection, and cancer [28,29]. However, constitutive expression of COX-2 also occurs in various organs, and activity of COX-2 in HeLa cells has been reported [30,31]. By its action, celicoxib will reduce the conversion of arachidonic acid to prostanoids and thus can be expected to increase arachidonic acid levels in the phospholipidome. This was not obvious from the PCA plot in which a celicoxib-specific phospholipidome was visible (Figure 2C,D), as polyunsaturated phospholipids are found scattered throughout the loading plot (Figure 2D). However, since PCA is designed to optimally reflect the variance in the entire dataset, a clearer celicoxib effect may be observed when only those samples and the control incubations are plotted. From the heatmap of relatively abundant species as depicted in Figure 3A, it can be concluded that arachidonic acid containing species (e.g., PE 38:4 and PE 36:4) are not particularly affected, as the log ratio in abundance of these lipids between control and celicoxib treated cells is close to zero (hence the ratio close to one).

#### 3.2.3. Interfering with Fatty Acid Metabolism by Orlistat, and Etomoxir

Orlistat, C75, and etomoxir all interfere with fatty acid metabolism and should be expected to have an effect on the phospholipidome of cultured cells. The pivotal role of fatty acid synthase (FASN) in cancer pathogenesis has led to a great interest in these drugs as anti-tumor candidates but a lipidomic characterization of their effects is lacking [32,33]. The drug C75 is a potent semi-synthetic inhibitor of three different domains of FASN: the β-ketoacyl synthase-, the thioesterase- as the enoyl reductase domain [32,34]. In our experiments, we found no effect of C75 on the cellular lipidome of HeLa cells (Figure 2A), despite the fact that the 20 µM concentration we used in our experiment has been shown to be effective in other cell lines [35,36]. One explanation could be that FASN is not active under our cell culture conditions. The cell culture medium contains an ample supply of fatty acids, either as albumin bound free fatty acids or esterified in lipoproteins. Under these conditions, de novo synthesis by FASN is not expected. Inactivity of FASN is supported by the fact that the inhibitor of fatty acid oxidation, etomoxir, does lead to an altered phospholipidome (Figure 2A,C). Simultaneous de novo synthesis of fatty acids and their degradation by β-oxidation is prevented by inhibition of acyl import into mitochondria by malonyl-CoA, the first product in the fatty acid biosynthetic path [37]. Since malonyl-CoA and etomoxir have the same target (carnitine palmitoyltransferase or CPT-1) and an effect of etomoxir is observed, mitochondrial import, or fatty acids is active under our experimental conditions. By extension, it can be concluded that FASN is not active, thus explaining the lack of a C75 lipotype. This lack of lipotype is also evident from Figure 3, as no lipid species have a log ratio clearly distinct from zero in the C75 samples. Interestingly, phosphatidylglycerol (PG) 34:1, a typical mitochondrial lipid species, is depleted (to 49% of control levels) after etomoxir exposure. Cardiolipin (CL), another typical mitochondrial lipid species was hardly affected.

Orlistat is also a potent FASN inhibitor but in contrast to C75, acts on the thioesterase domain of the enzyme [38,39]. Besides FASN, orlistat also inhibits (gastric and pancreatic) lipases and for this activity, orlistat is often prescribed as an anti-obesity drug [40]. In our experiments, orlistat administration led to a clearly distinct lipidome (Figure 2A,C). Considering the fact that our C75 and etomoxir experiments suggested FASN to be inactive, the effects of orlistat on the HeLa lipidome could be due to its broad lipase inhibitor activity [40]. Orlistat inhibition gives a clear illustration of the complexity in which the lipidome is given shape (Figure 3). Some polyunsaturated phosphatidylethanolamine (PE) species levels are strongly decreased (e.g., PE 36:5, PE 36:4, PE 38:4) whereas levels of others are unaffected (PE 38:6, PE 40:6, PE 40:7). Mono- and di-unsaturated PE species are unaffected (PE 34:1 and PE 36:2), but the levels of their phosphatidylcholine PC counterparts (PC 36:1, PC 36:2) are strongly up.

#### 3.2.4. Inhibitors of Autophagy: Spautin and Bafilomycin

Autophagy, the turnover of selected cytoplasmic components and organelles, can be an important provider of building blocks in lipid metabolic pathways. We therefore investigated the effect of two drugs with autophagy inhibiting properties on the lipidome of HeLa cells. Spautin (specific and potent autophagy inhibitor) selectively inhibits autophagy by promoting the degradation of Beclin1 through the proteasomal pathway [41]. Because of the increased degradation, Beclin1 is missing as an essential part of the multi-protein complex that initiates the formation of the autophagosome [42,43]. Indeed, spautin has a clear effect on the lipidome of HeLa cells (Figure 2A,C) as can be concluded from the clear separation of spautin- and control samples. The most prominent differences between spautin and control samples are reflected in principal component 2. Lipids with highest (absolute) loading values on principal component 2 (i.e., PE 36:2, PE 34:1, PE 36:1, and the polyunsaturated PE species at the bottom of Figure 2B) are the most likely candidates for relevant changes based on the PCA loadings plot (Figure 2B). This is indeed confirmed in the lipid-inhibitor heatmap (Figure 3) in which these lipids are represented by green squares. Somewhat surprising is the fact that the levels of the two ‘mitochondrial’ lipids (PG and CL) are not up, although mitochondrial degradation by autophagy is inhibited [44].

Bafilomycin is a macrolide antibiotic isolated from the Streptomyces species, and an inhibitor of vacuolar H + ATPase (V-ATPase) [45]. The drug has been frequently used in the study of autophagy as it inhibits the fusion between autophagosomes and lysosomes, preventing autophagic degradation [46,47]. Bafilomycin had a very strong influence on the HeLa lipidome as can be concluded from the great distance between control- and bafilomycin samples (Figure 2A). Surprisingly, the effects of bafilomycin and spautin on the lipidome appeared to be reciprocal, as these inhibitors are in opposing quadrants of the PCA score plot, with control samples near the origin. High SM content contributes to high PC-1 values (Figure 2B) and indeed a 98% increase in total SM was observed in the bafilomycin samples (*p* < 10^-8^). In contrast, a 16% decrease in SM was observed in the spautin samples (*p* < 10^-3^). Also, in the lipid-inhibitor heatmap (Figure 3), little resemblance between the effects of spautin and bafilomycin is observed. This clearly demonstrates that, although both drugs inhibit autophagy, their different modes of action results in very distinct cellular phenotypes.

#### 3.2.5. Rosiglitazone, a Thiazolidinone Drug that Acts as a Peroxisome Proliferator Activity Receptor γ Agonist

The principal actions of rosiglitazone are thought to be alterations in gene expression mediated by peroxisome proliferator activity receptor γ (PPARγ) [48,49]. Rosiglitazone is prescribed to reduce insulin resistance in patients with type 2 diabetes, as PPARγ is an important regulator of lipid and glucose metabolism [50]. Gene targets of PPARγ include fatty acid-binding, -transport, and -translocase proteins and an effect of rosiglitazone on the cellular lipidome may therefore be expected [51]. Indeed, our experiments show a clear effect of PPARγ activation by rosiglitazone on the HeLa lipidome (Figure 2A,C). Notably, changes in lipidome were reflected in PC-2 and PC-3, but hardly in PC-1. The SM lipid class, of which species have a large loading on PC-1, indeed did not change (*p* = 0.7). The abundant lipid species PE 34:1 and PE 34:2 were both much more abundant after inhibition by rosiglitazone (Figure 3). Since the enzyme that catalyzes the final step in PE synthesis has a preference for mono- and di-unsaturated species, this suggests that rosiglitazone either upregulates de novo PE synthesis or inhibits the subsequent remodeling of newly synthesized PE species [52,53].

### 3.3. Correlations between Lipids

**Figure 4:**
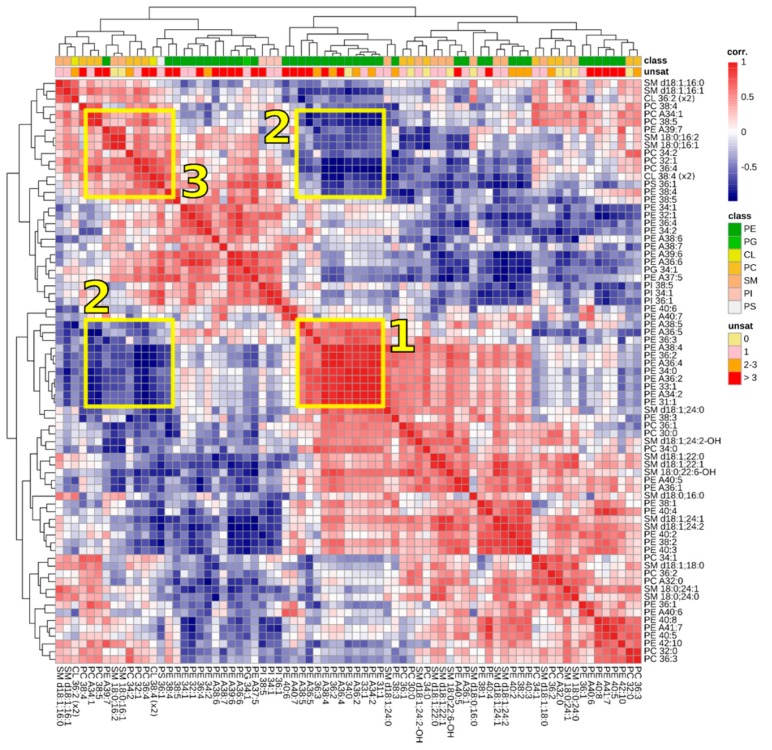
Heatmap of lipid-lipid correlations. The 75 most abundant lipid signals were ordered using hierarchical clustering. Correlations between lipid species are colored from strong positive correlation (red), to no correlation (white), to negative correlation (blue). The boxed areas 1–3 are discussed in the main text.

The molecular species composition of cells is the result from the action of many enzymes. These enzymes have, to various degree, a preference for lipid classes or fatty acyl composition [19,54,55]. Apart from chemical composition, also exposure/availability of lipid substrates to metabolizing enzymes contributes to the steady-state lipidome. In our experiments, we have interfered with various pathways of lipid metabolism. It is interesting to analyze which lipids behave similarly during these experiments, as these lipids apparently are synthesized and degraded at similar rates. We therefore constructed a correlation matrix, in which the level of each lipid was plotted against that of any other lipid in all experiments. For each combination of lipids, we then calculated the corresponding correlation coefficients of linear regression analysis and plotted the correlation values as a heatmap (Figure 4). In this figure, lipid species behaving identical in experiments (i.e., going up and down at identical rates) are colored red, whereas species in blue had opposite behavior: high levels of one species corresponded with low levels of the other and vice versa. White intersections indicate no correlation between the two lipid species.

Within the HeLa lipidome, clusters of lipid species with surprisingly high correlation were found. One of these clusters, boxed and marked ‘1’ in Figure 4, consisted entirely of PE species, either from the ether- or diacyl subclass. These PE species did not share a common degree of unsaturation nor did they have similar acyl chain lengths. Common PE species such as PE 36:2 and PE 36:3 were part of this cluster, whereas other abundant PE species (e.g., PE 36:1, PE 34:1, PE 36:4) were not. It is attractive to speculate that this cluster reflects a particular intracellular membrane or a fraction thereof. A clue about which membrane fraction this might be, can be derived from another cluster of lipids that correlates negatively with the aforementioned PE species. This cluster is boxed and marked ‘2’ in Figure 4. Because of the symmetry of the heatmap, the cluster is depicted twice. This negatively correlating cluster contained lipids typical for plasma membrane (SM species) or mitochondria (CL). This suggests that cluster ‘1’ does not represent these membranes. The suggestion that cluster ‘2’ consists of lipids from different membranes is supported by the fact that correlations between lipids in cluster ‘2’ (boxed and indicated by ‘3’ in Figure 4) are much weaker than in cluster ‘1’. Unfortunately, literature does not yet provide clear data off subcellular lipidomics that can be universally used to shed a light on the identity of the lipid clusters of Figure 4 [56].

## 4. Discussion

Interpretation of lipidomic data can be extremely challenging and is exemplary for the multidisciplinarity of contemporary ‘omics’-technology in which cell biologists, biochemists and bio-informaticians collaborate. This is in part due to the fact that there is no obvious relationship between abundant lipid species and metabolic- or signaling pathways as is the case in proteomics or genomics. Furthermore, phospholipids are composed of a combination of several variable building blocks and this gives a sheer endless list of possible lipid species. On the other hand, lipid composition is directly related to cell function. For instance, the extremely high content of polyunsaturated phospholipids in sperm cells is required for the intra- and intercellular membrane fusion events in fertilization, whereas the exceptionally high content in fully saturated species is required for the surface tension lowering effect of lung surfactant [20,57]. The complex lipidome can thus be a sensitive and versatile indicator of cell health or pathology, but only after we have learned to fully understand lipid composition is its proper context.

In this manuscript, we have demonstrated a high-throughput method that can help to start to understand the consequences of perturbation of normal lipid homeostasis by specific drugs. As a proof of principle, we have shown highly reproducible and specific changes for nine different drugs in various lipid pathways. Given this high reproducibility, one might choose to perform incubations in duplicate instead of quadruplicate and increase the number of screened drugs without increasing work load or losing sensitivity. With an LC-MS run time of only four minutes, an instrument can process 360 samples per day. Valuable and often essential compound library screening has thereby come within reach for every lipidomics laboratory [58].

In the set of inhibitors investigated here, we were rather surprised by the specificity in lipidome changes that were induced. Apart from C75, which did not induce any new lipid phenotype, none of the inhibitors overlapped with control samples or another inhibitor in a PCA of the lipid data. This demonstrates a unique mode of action of each inhibitor. Initially, one could expect at least the two sphingolipid biosynthesis inhibitors to be much more similar, as they both are considered to be specific inhibitors of the same pathway and have considerable structural homology. In that respect, it is remarkable to see that the main difference between these two drugs was their distinctive effect on specific glycerophospholipid species. This illustrates the complex way in which lipid species relate to each other and our current lack in understanding these relationships.

Our data also comprise a warning. Lipid–protein interplay is key in the regulation of many cellular processes [59,60]. By consequence, inhibitors that induce large compositional changes in the cellular lipidome, may alter normal activity of untargeted processes as a secondary effect. Spautin and bafilomycin are both widely used as autophagy inhibitors, but the dramatic effects of bafilomycin on many more lipid species, suggests that bafilomycin is more prone to induce unforeseen secondary effects through lipid–protein alterations.

A valuable application of the high-throughput pipeline presented here, lies in thorough characterization of drugs. Side effects of drugs can be detrimental but also beneficial. For instance, the drug orlistat that was used in this study, was discovered to have anti-tumor activity through FASN inhibition as a beneficial side effect, next to its known activity as lipase inhibitor [61]. Similarly, many FDA-approved drugs have multiple targets, depending on the concentration at which the drug is administered [62]. These novel applications for existing, approved drugs are highly interesting for pharmaceutical companies and patients alike, as costly toxicological studies can be avoided. High-throughput lipidomic analysis with PCA such as described here, can be used to investigate dose–response relationships. From the same data, it can be inferred at what concentration other molecular targets become involved. This may be observed in a PCA score plot as samples of increasing inhibitor concentration suddenly moving in another direction above the threshold of this secondary drug target.

Apart from the characterization of drugs, the same strategy maybe applied for the classification of cell lines or tumors. Reprogramming of lipid metabolism in tumor development can occur in a number of ways and may make these cancers more or less susceptible to specific drugs [63,64]. Once we are able to interpret the response of the lipidome to drug challenges, in vitro drug screens of cancerous cell cultures from patients may become a valuable asset in personalized medicine. If not conclusive, at least these screens can contribute to the identification or rejections of suitable targets.

Taken together, we demonstrate unique phospholipid responses to various drugs in HeLa cells. We present ways to reduce data complexity and subsequently visualize drug responses so drug–lipid relationships become better understandable. We feel that our data contributes to the understanding of the effects of the widely used inhibitors we investigated. Furthermore, we outlined additional ways in which our methodology can be applied and we trust that this will be valuable to many. In the end, we think that lipidomics will prove to be the -omics tool of choice when it comes to understanding lipid metabolic processes and abnormalities.

## Supplementary Materials

**Supplementary table 1A:** Top 100 of LCMS signal contributions of unique lipid species. APCI generated ions were detected in a linear ion trap and annotated by their mass to charge ratio and retention time. In the case of co-eluting species with identical n. **Supplementary table 1B**: *p*-Values compared to CTRL. Values are not corrected for multiple comparison. **Supplementary table 2:** Lipid species detected in HeLa cells by negative mode electrospray ionization on a high-field orbitrap MS following hydrophilic interaction liquid chromatography. Lipids are annotated by their experimentally determined mass to charge ratio and retention time. Note that PC, HexCer and SM species are detected as their formate adducts. An 'A' in the lipid name indicates an ether species. Sphingosine (d18:1) was assumed as the sphingoid base, only when no match was found, sphinganine (d18:0) was assumed. **Supplementary methods:** MS acquisition parameters.
